# Mechanical Properties and Diffusion Studies in Wax–Cellulose Nanocomposite Packaging Material

**DOI:** 10.3390/ijms23169501

**Published:** 2022-08-22

**Authors:** Chandra Mouli R. Madhuranthakam, Shannon Q. Fernandes, Antonella Piozzi, Iolanda Francolini

**Affiliations:** 1 Chemical Engineering Department, Abu Dhabi University, Abu Dhabi 59911, United Arab Emirates; 2 Chemical and Biomolecular Engineering Department, Lehigh University, Bethlehem, PA 18015, USA; 3 Department of Chemistry, Sapienza University of Rome, 00185 Rome, Italy

**Keywords:** nonacosan-10-ol, nonacosan-5,10-diol, cellulose nanocomposite, gas diffusion, molecular simulations, glass transition temperature

## Abstract

This article focuses on the study related to the estimation of packaging material properties of cellulose–wax nanocomposite using molecular dynamics simulation (MDS). Cellulose based packaging material is gaining lot of importance due to its good material properties and low cost. Cellulose with small amount of plant-derived wax (nonacosane-10-ol and nonacosane-5,10-diol) offers higher mechanical strength and modulus of elasticity compared to the conventional synthetic polymer materials. In this article, in addition to the estimation of mechanical properties, the thermal stability of the proposed ecofriendly cellulose–wax composite is evaluated by estimating the glass transition temperature which essentially provides critical information on the glassy state and rubbery state of this biopolymer. The glass transition temperature of this composite changes significantly compared to that of pure cellulose (which also suffers from poor mechanical strength). Transport properties such as diffusion volume and diffusion coefficient of oxygen, nitrogen, and water are estimated using the results obtained from MDS. The diffusion coefficients of these species within the cellulose–wax composite are analyzed using the diffusion volume and interaction energies of these constituents with the wax and cellulose.

## 1. Introduction

Packaging material is predominantly selected based on its permeability to gases, water vapor, oil, grease, heat, light, and microorganisms which can directly or indirectly affect the product quality and shelf life. Different polymer materials, either synthetic or natural, have been engineered to confer antimicrobial features [1,2,3]. Packaging material made of biopolymers is gaining more importance to overcome the problem of disposability and degradability of synthetic polymer-based packaging material. With food packaging materials, consumers always prefer and demand use of natural biomaterials compared to synthetic materials. There is a huge gap in the differences between the properties of synthetic polymer materials and natural biomaterials. While it is not possible to completely replace polymer-based packaging materials, extensive research is being done to use blends of natural biomaterials with polymers which also addresses the issue of minimizing plastic footprint and offers sustainable solutions in some scenarios. Among several packaging materials, cellulose which is the most abundantly available biopolymer in nature is being used along with other additives in many commercial and food packaging applications. Cellulose without any modification or additives is not used as packaging materials but is used in making paper and boards. In addition to being biodegradable, by selectively adding additives, cellulose-based packaging materials can offer good barrier properties, biocompatibility, antioxidant activity, antimicrobial properties, and excellent mechanical properties [3,4,5,6,7]. Cellulose along with other biopolymers can also assemble into different forms and shapes such as powders, films, gels, and solutions which enables to be used in versatile packaging material meant for different purposes [8,9]. Additives are in general added to cellulose not only to achieve desired end properties of the packaging material but also to reduce the cost of the composite material. In addition to achieving good physical and barrier properties, some of the most recent objectives in adding additive are to preserve freshness of the product, increase shelf life of the product, and also help in monitoring the quality changes in food products by using the so-called intelligent packaging ingredients [10,11]. While cellulose is blended with other synthetic polymers such as polyethylene (PE) and polypropylene (PP), it is also used in stand-alone packaging material synthesis along with other active and intelligent additives [12,13,14,15]. Fernandes and Madhuranthakam [16] showed from molecular simulations that cellulose coated with natural wax material consisting of nonacosane-10 ol and nonacosane-5,10 diol (called as adulose) enhanced the mechanical properties when used for packaging material. In addition to having high Young’s modulus and ultimate stress, adulose is also superhydrophobic, which enables its use as a packaging material in many different applications. The additives used in the formation of adulose are plant-derived wax [17,18], which when combined with cellulose makes this composite to be a completely biomaterial-based sustainable packaging material.

In this article, mechanical properties, thermal stability, and diffusion of certain species in adulose are studied using molecular dynamics simulations (MDS). MDS is an effective tool for predicting and estimating the barrier properties, mechanical and thermal behavior of materials. Using the results obtained from MDS, mechanical properties are studied by conducting stress strain simulations while thermal stability is studied by estimating and evaluating the glass transition temperature (T_g_). Diffusion of oxygen, nitrogen, and water molecules in adulose is studied by calculating the diffusion coefficient and fraction free volume (FFV) which are important barrier property attributes. The results from this article complement the findings on adulose from Fernandes and Madhuranthakam [16] which in turn gives a complete understanding of the role of nonacosane-10 ol and nonacosane-5,10 diol in cellulose.

## 2. Results and Discussion

**Figure 1:**
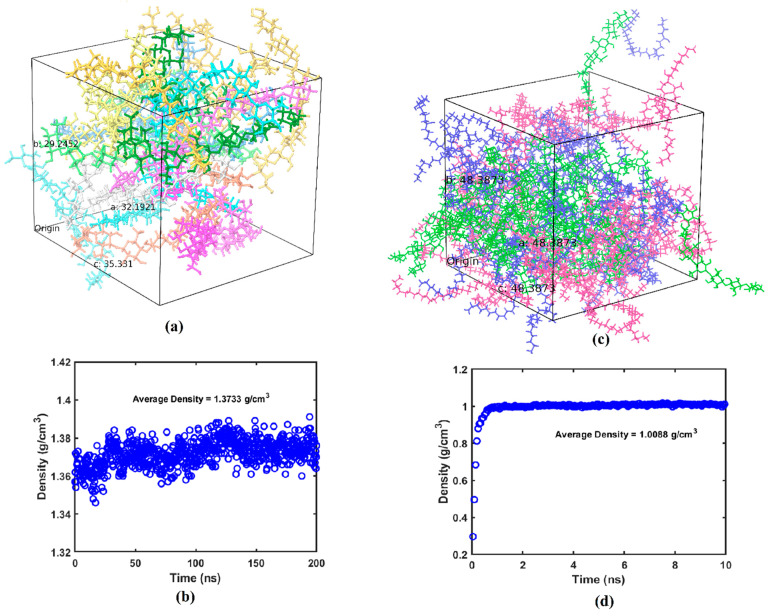
(**a**) Equilibrated amorphous cellulose (each chain is represented by a different color), (**b**) density-time profile for cellulose, (**c**) equilibrated adulose (cellulose chains are shown in green, nonacosane-10 ol molecules are shown in purple, nonacosane-5,10 diol molecules are shown in blue), (**d**) density-time profile for adulose.

The system with cellulose chains is relaxed and equilibrated for 200 ns while the systems with the two different types of wax are relaxed and equilibrated for 10 ns. Cellulose has longer chain length compared to the wax molecules due to which MDS for cellulose is performed for longer simulation time. A reasonable value for equilibration time is also decided based on the constant density/volume profiles obtained in these simulations. Figure 1a shows the relaxed and equilibrated amorphous cellulose system and Figure 1b shows the density profile with respect to equilibration time. Similarly Figure 1c,d show the equilibrated adulose system and density profiles respectively.

The density of the equilibrated amorphous cellulose is obtained to be 1.3733 g/cm^3^. This is in very good agreement with the range of 1.34 to 1.39 g/cm^3^ reported by Mazeau and Heux [19]. Both nonacosane-10-ol and nonacosane-5,10-diol are derivatives of nonacosane which has a density of 0.808 g/cm^3^. The density of nonacosane-10-ol is 0.840 g/cm^3^ and the density of adulose is obtained as 1.008 g/cm^3^ from the MDS. Both nonacosane-10-ol and nonacosane-5,10 diol are plant-derived waxes that have least mechanical strength but offers advantages with respect to achieving enhanced properties when used as a filler in cellulose and polyethylene-based composites [16,20]. Djokovic et al. [21] found that a small addition of oxidized Fisher-Tropsh wax (which is a synthetic chemical) can improve the mechanical properties of polyethylene.

**Figure 2:**
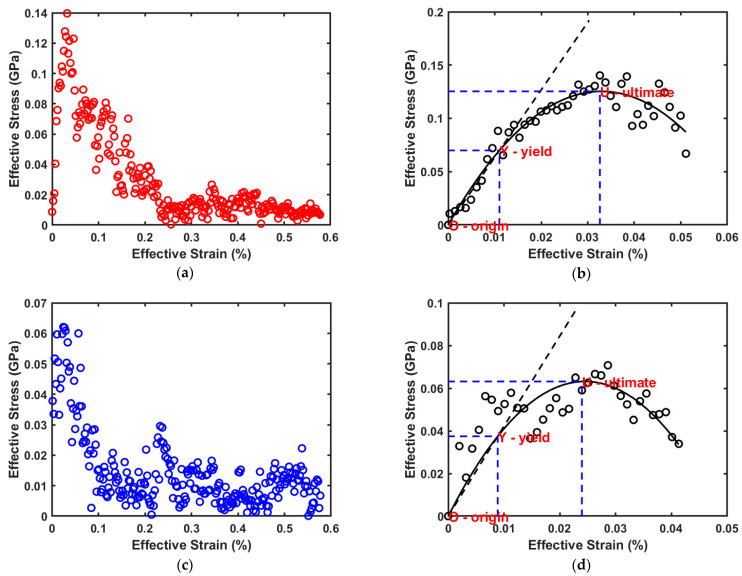
(**a**) Effective strain vs. effective stress data for cellulose. (**b**) Stress–strain model fit for cellulose. (**c**) Effective strain vs. effective stress data for adulose. (**d**) Stress–strain model fit for adulose.

Figure 2a,b show the results obtained from the stress strain simulations for cellulose, while Figure 2c,d show the corresponding results obtained for adulose. Using a nonlinear least squares MATLAB program, a second order polynomial (with zero intercept) is fit to the stress–strain data obtained from the molecular simulations. The ultimate stress and ultimate strain (corresponding to the point U in Figure 2b,d are obtained from the corresponding maximum values for the fitted polynomial. The yield stress and yield strain that constitutes the elastic limit represented by the point Y in Figure 2b,d is found by fitting a straight line (with zero intercept passing through the origin “O”) to the portion of the second order polynomial beyond which the slope does not change. The Young’s modulus of the material is obtained from the slope of this fitted line. This procedure can be easily understood by referring to the stress–strain curves obtained for cellulose and adulose as shown in Figure 2b,d respectively. The summary of the mechanical properties for cellulose and adulose corresponding to the Figure 2b,d is shown in Table 1.

Table 1 clearly shows that the elastic modulus, ultimate stress, and yield stress of adulose are less than those of cellulose. Similarly, the yield strain and ultimate strain of adulose are also comparatively less than that of cellulose. Fernandes et al. [16] showed that the mechanical properties of adulose were almost similar or even better than that of cellulose under conditions of the cellulose being coated with the chains of nonacosane-10-ol and nonacosane-5,10-diol. There are two important differences that are noteworthy in comparing the results from Fernandes et al. [16] and the results obtained in the current work. The first difference is Fernandes et al. used linear chains of cellulose and the waxes in the MDS which is different from the more practical scenario of tangled chains and amorphous cellulose used in this article. The second difference is that Fernandes et al. used a layer by layer of two waxes around the cellulose chains while in this work a nanocomposite of well mixed amorphous cellulose chains with the waxes is used. On the other hand, with 29% of nonacosane-10 ol alone with polyethylene blend, Madhuranthakam et al. [20] showed that enhanced mechanical properties were obtained. However, the elastic modulus of adulose is observed to be greater than that of polyethylene or polyethylene–wax composite which makes adulose to be a good choice when targeted to be used as a packaging material with less flexibility. At the same time, adulose is more elastic than cellulose which facilitates use of adulose in at least some of the packaging materials in lieu of the synthetic non-biodegradable plastic packaging materials. Adulose derives maximum mechanical strength from the cellulose chains, and it is observed that other desirable properties for adulose are obtained as explained in the following sections.

While the strength of the materials considered in this study is assessed by analyzing its mechanical properties, the stability of these materials is further investigated by conducting thermophysical simulations. Glass transition temperature (T_g_) is calculated from these simulation results. T_g_ values of amorphous cellulose and adulose are obtained by using a piece-wise bilinear fit to the temperature versus specific volume data obtained from the thermophysical simulations.

**Figure 3:**
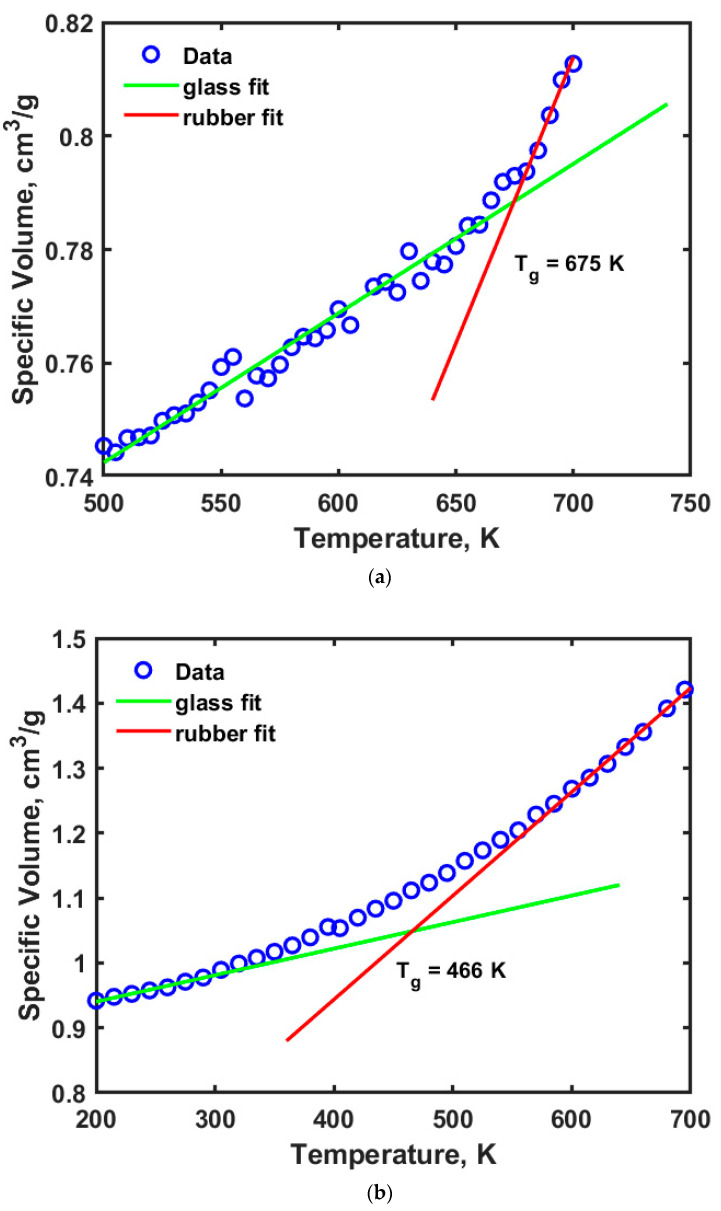
Temperature versus specific volume profiles for (**a**) cellulose (**b**) adulose.

Figure 3a,b show the profiles for the temperature versus specific volume obtained from the thermophysical simulations for cellulose and adulose respectively. Figure 3a shows that the T_g_ of cellulose is 675 K while T_g_ of adulose is obtained as 466 K. Cellulose exhibits different transitions based on its crystallinity and amount of water associated with it. Within cellulose, there are α-cellulose, β-cellulose, and amorphous cellulose types and each of them have a different range of T_g_ values. Wang et al. [22] reported that T_g_ of amorphous cellulose is 448 K using simulations while Szczes’niak et al. [23] obtained a T_g_ of 493 K from experiments where cellulose powder was used. Mazeau and Heux [19] obtained a T_g_ of amorphous cellulose to be 650 K and suggested an addition of 40 K due to the time scale at which experimental measurements were made. In our study, a 200 ps dynamics corresponds to approximately frequencies in the order of 1 MHz. The results obtained in this study are in close agreement with Mazeau and Heux [19] based on the MDS parameters used for amorphous cellulose. Glass transition temperature is an important thermophysical property which is attributed to the flexibility of a polymer due to the movement of the backbone chains which in turn occurs due to rotational and translational motion. This movement further leads to the generation of free volume or unoccupied space with an opposite effect i.e., higher free volume leads to lower T_g_ values and vice versa. For the amorphous cellulose studied in this work, a high value of T_g_ is observed. The free volume of cellulose is obtained by using a molecular probe with certain radius R_P_ that moves on the van der Waals surface. The fraction free volume, FFV is defined according to Equation (1).

(1) $$\mathrm{FFV}=\frac{V_{F}}{V_{F}+V_{O}}$$

In Equation (1), V_F_ is the free volume of the polymer, V_O_ is the volume occupied by the polymer and the sum of V_F_ and V_O_ is the total volume of the polymer. The free volume estimation is also helpful in understanding the diffusion characteristics of different species in cellulose/adulose. With a probe radius of 0.1 Å, the FFV for cellulose is obtained to be 28.24%, while for adulose it is 75.47%. The low value of T_g_ for adulose can be understood from the perspective of FFV values obtained from MDS. By adding nonacosane-10-ol and nonacosane-5,10 diol to the cellulose, with a portion of these molecules occupying the free space in cellulose, it is observed that there is a huge increase in the volume. The volume of cellulose is 32,977 Å^3^ while volume of adulose is 113,310 Å^3^. From the thermophysical results, with the T_g_ of adulose being far less than that of cellulose it is clear that adulose can be used as packaging material for a wide range of applications.

**Figure 4:**
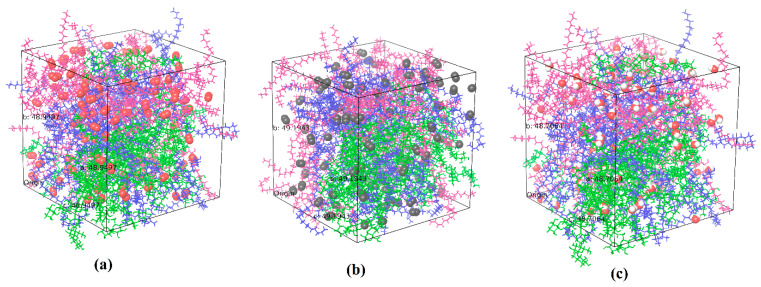
Equilibrated and relaxed adulose with (**a**) oxygen, (**b**) nitrogen, and (**c**) water molecules (red spheres are oxygen, grey spheres are nitrogen, red sphere with two white spheres are water molecules).

**Figure 5:**
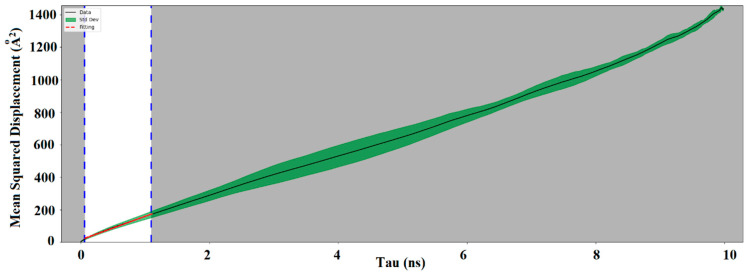
MSD curve obtained for adulose with oxygen molecules (black solid line is the data, green region is the standard deviation, and the red line is the fitting).

The barrier properties of adulose is further assessed by studying the diffusion of oxygen, nitrogen, and water using MDS. Oxygen, nitrogen, and carbon dioxide are in general used in modified atmosphere packaging. Diffusion rates of these species are very important to decide on the type of application in which adulose can be used as a packaging material. The requirement of high or low diffusion rates of these species strongly depends on the characteristics of the material stored and also its intended shelf life or end-use applications. If the packaging material is used for storing fresh food then a low diffusion or permeability rate of oxygen is desired as it can reduce the oxygen pressure inside due to which the shelf life of the product increases. Self-diffusion of oxygen simulations showed that 100 molecules of oxygen would represent similar to a bulk oxygen system [20]. With respect to the number of water molecules used, the SPC/E model gives reliable diffusion rates very close to the experimentally observed values independent of the number of water molecules used in the simulation [24]. In this study, 100 molecules of oxygen, nitrogen, and water are used to estimate the diffusion coefficient in cellulose and adulose. The MSD of the corresponding molecules are obtained from which the diffusion coefficient is estimated. More accurate estimation is obtained using the linear portion of the MSD. Figure 4a–c shows adulose with oxygen, nitrogen, and water molecules respectively. In Figure 4, tau is the simulation time difference and the linear portion of the MSD corresponds to tau values in the range of 0 to 0.99 ns. Figure 5 shows the MSD obtained for adulose with oxygen system (similar curves are obtained for adulose with nitrogen and water systems).

The diffusion coefficients at 300 K and 1.01325 bar obtained from the MDS are shown in Table 2 along with the FFV percentages (calculated according to Equation (2)) using a probe radius of 0.1 Å.

**Figure 6:**
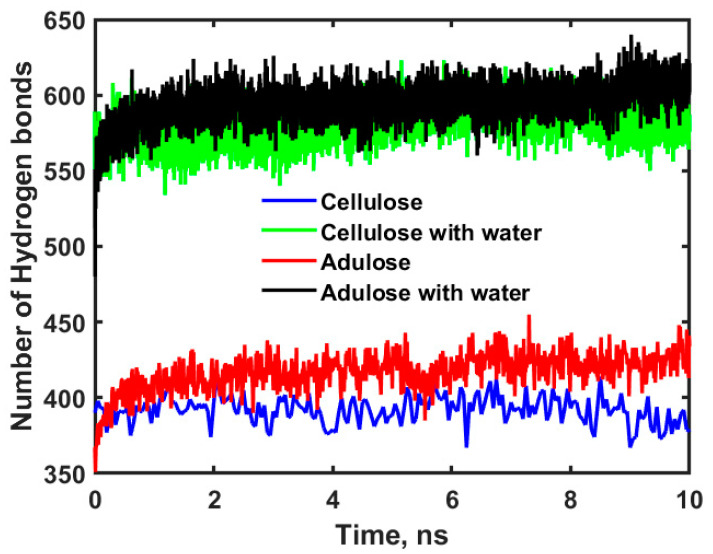
Time versus number of hydrogen bonds for cellulose, cellulose with water, adulose, and adulose with water systems.

The experimental diffusion coefficients of oxygen, nitrogen, and water in cellulose depends on several factors such as solubility, permeability, pressure, temperature, and source from which cellulose is extracted. The diffusion coefficients for oxygen, nitrogen and water in cellulose obtained in this work are comparable to the values reported by Minellia et al. [25] and Wang et al. [26]. The diffusion coefficients obtained for oxygen, nitrogen, and water in adulose systems are greater than those obtained for cellulose systems. This can be understood from the FFV values estimated from MDS and shown in Table 2. The atomic radius of oxygen and nitrogen are 60 and 65 pm due to which the FFV values obtained for them in both cellulose and adulose are almost same which in turn resulted in obtaining similar diffusion coefficient values. The diffusion coefficient of oxygen in adulose is 2.39 × 10^−10^ m^2^/s which is 50% less than that of diffusion coefficient of oxygen in polyethylene (5.062 × 10^−10^ m^2^/s) [20]. This makes adulose to be a competing candidate for the packaging material with enhanced barrier properties compared to that of polyethylene-based packaging material. The FFV for water is observed to be less than that of oxygen or nitrogen in cellulose/adulose due to an increase in the hydrogen bonds formed by water molecules with cellulose. A hydrogen bond is defined as the attraction of a covalently bonded hydrogen atom with another electronegative atom. In this study, a hydrogen bond is defined geometrically as having a hydrogen-acceptor distance of less than 2.8 Å, minimum donor angle to be 120° and minimum acceptor angle to be 90°. The number of hydrogen bonds directly or indirectly affect not only the mechanical properties but also the anti-aging performance [27]. Figure 6 shows the profiles for hydrogen bonds obtained for cellulose, cellulose with water, adulose, and adulose with water systems.

**Figure 7:**
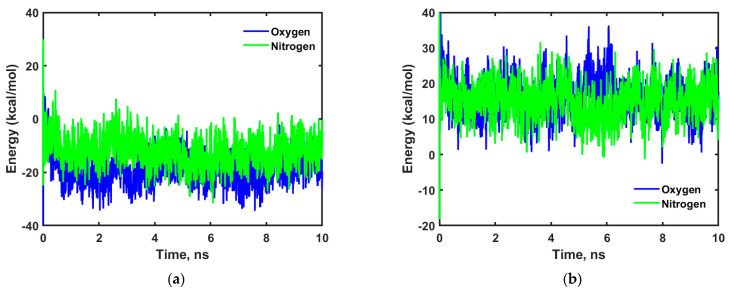
Time versus interaction energy for oxygen and nitrogen in (**a**) cellulose and (**b**) adulose.

The statistical average of the number of hydrogen bonds for cellulose, cellulose with water, adulose, and adulose with water systems is found to be 392, 581, 417, and 595 respectively. Further the higher diffusion coefficients of oxygen and nitrogen in cellulose can be understood by estimating the interaction energy. Interaction energy (E_int_) indicates the intensity of the interaction between the diffusion molecule and the main chains (cellulose or wax molecules). It is calculated by using the Equation (2).

(2) $$E_{\mathrm{int}}=E_{\mathrm{total}}-E_{\mathrm{cellulose}}-E_{\mathrm{wax}}-E_{i}$$

where E_total_ is the total energy of the system, E_cellulose_ is the energy of the cellulose chains, E_wax_ is the energy of the nonacosane-10-ol and nonacosane-5,10 diol and E_i_ is the energy of the diffusing species i (oxygen or nitrogen or water in this case). Any species “i” will have stronger interaction if the corresponding E_int_ value is very high with a negative magnitude. A higher energy barrier has to be overcome for molecules with high E_int_ values which in turn also means that their corresponding diffusion coefficients will be low. Figure 7 shows the energy profiles obtained for oxygen and nitrogen in cellulose and adulose.

As shown in Figure 7a,b, oxygen and nitrogen have high negative interaction energies in cellulose compared to that of adulose. This means oxygen and nitrogen have strong interaction to cellulose chains compared to adulose which also explains the higher diffusion coefficient observed in adulose compared to that of cellulose.

## 3. Methods and Materials

All molecular dynamics simulations were performed using the Material Science (MS) Suite version 4.4.135 of Schrödinger 2022-1 release (Schrödinger, LLC, New York, NY USA) with OPLS4 force field [28]. The amorphous cellulose chains (the repeating unit for cellulose homopolymer is beta-D-glucose and 12-mers are used in this simulation), nonacosan-10-ol, nonacosan-5,10 diol, oxygen, nitrogen and water molecules chemical structures were drawn using the 2D sketcher which were further converted to 3D using the MS Maestro interface. Adulose with 95% cellulose, 3% nonacosan-10-ol, and 2% nonacosan-5,10-diol (all weight percent) was built using the Disordered System builder in the MS Suite. A unit cell with 5 nm × 5 nm × 5 nm was used. Twenty-four chains of cellulose each consisting of 149 atoms were embedded with 48 molecules of each of the waxes (nonacosan-10-ol and nonacosan-5,10 diol). Using the Multi-Stage Simulation workflow in MS suite, all the structures were initially relaxed and equilibrated for 200 ns. The relaxation and equilibration involved conducting MD simulation for 10 ns initially at 300 K and 1.01325 bar with NPT ensemble followed by Brownian minimization for 100 ps and finally MD simulation for 200 ns with NPT ensemble at 300 K and 1.01325 bar. The analysis of bulk properties for the final system was performed after equilibration. The stress strain calculations were performed using the option of pure uniaxial condition, with a strain rate of 1.0 × 10^8^ s^−1^ and using a strain step size of 0.001 for 1000 steps. The stress–strain simulations were run for a maximum strain of 0.9. The corresponding results are used for estimating the Young’ modulus, yield stress, yield strain, ultimate stress, and ultimate strain. In the simulation protocol, a simulation time of 10 ps with a time step of 2.0 fs is used and a trajectory recording interval of 5 ps is set at a temperature of 300 K. The number of hydrogen bonds and the interactive energies for all scenarios are estimated and used to understand the behavior of the cellulose nanocomposite. Simulations related to thermophysical properties for evaluating glass transition temperature were conducted by cooling the cellulose and adulose systems from 700 K to 200 K in steps of 5 K constrained to convergence from each previous step. These simulations were performed for 10 ns at a pressure of 1.01325 bar, for three maximum cycles and corresponding to a 5% convergence. A trajectory of temperature versus specific volume was obtained from all thermophysical property simulations. Using a bilinear fit for the rubbery region and glassy region, the corresponding glass transition temperature was obtained for cellulose and adulose. Barrier properties of the cellulose nanocomposite are studied and estimated by conducting diffusion simulations. The main diffusion species considered in this study are oxygen, nitrogen and water. Einstein’s method of estimating the diffusion coefficient from the mean square displacement (MSD) curve was used [29]. According to Einstein’s equation, the diffusion coefficient (D) of a species is calculated as shown in Equation (3).

(3) $$D=\frac{1}{6N}\underset{t\to \infty }{\lim}\frac{d}{\mathrm{dt}}\overset{N}{\underset{i=1}{\sum }}\langle {\left[r_{i}\left(t\right)-r_{i}\left(0\right)\right]}^{2}\rangle$$

where N is the number of molecules of that species, r_i_(0) and r_i_(t) are the initial position coordinate and position coordinate of particle i at any time, t respectively.

## 4. Summary and Conclusions

Cellulose-based nanocomposite that consists of plant-derived waxes, nonacosane-10-ol and nonacosane-5,10-diol was simulated to assess its potential for using as packaging material. Molecular dynamics simulations were conducted from which different properties of this material were obtained. Mechanical properties simulations showed that adulose has Young’s modulus of 4.2248 GPa which is greater than that of polyethylene while less than that of pure cellulose. Other mechanical properties such as the ultimate stress, ultimate strain, yield stress, and yield strain were obtained for adulose. From the thermophysical property simulations, the glass transition temperature of adulose was found to be 466 K which is less than that of cellulose which was found to be 675 K. Addition of very small amount of waxes to cellulose led to a significant decrease in the T_g_ values. Furthermore, the amount of wax added to cellulose can be manipulated and optimized for obtaining T_g_ values that are amenable to process adulose and use it as a substitute for polyethylene packaging material. Barrier properties of adulose showed that it can be a potential candidate for using in packaging applications. The diffusion coefficient of oxygen, nitrogen, and water molecules through adulose were obtained to be 2.39 × 10^−10^, 2.45 × 10^−10^, and 1.61 × 10^−11^ m^2^/s respectively which are at least 50% less than that of corresponding diffusivities reported in polyethylene. It can be concluded that adulose is an ecofriendly sustainable packaging material with good mechanical, thermal, and barrier properties.

## Figures and Tables

**Table 1:** Mechanical properties of cellulose and adulose.

System	Elastic Modulus (GPa)	Ultimate Strain (%)	Ultimate Stress (GPa)	Yield Strain (%)	Yield Stress (GPa)
Cellulose	6.3391	0.0326	0.1253	0.011	0.07
Adulose	4.2248	0.024	0.0632	0.0089	0.0375
Polyethylene [13]	2.3792	0.1328	0.1878	0.0433	0.1030

**Table 2:** Diffusion coefficients and FFV for oxygen, nitrogen, and water in cellulose and adulose.

System	Diffusion Coefficient (m^2^/s)	FFV (%)
Cellulose-oxygen	8.51 × 10^−11^	26.31
Cellulose-nitrogen	8.35 × 10^−11^	28.88
Cellulose-water	8.2 × 10^−12^	24.94
Adulose-oxygen	2.39 × 10^−10^	30.67
Adulose-nitrogen	2.45 × 10^−10^	31.49
Adulose-water	1.61 × 10^−11^	30.33

## Data Availability

Not applicable.
